# Endophytic *Bacillus velezensis* strain B‐36 is a potential biocontrol agent against lotus rot caused by *Fusarium oxysporum*

**DOI:** 10.1111/jam.14542

**Published:** 2019-12-19

**Authors:** G.F. Wang, J.F. Meng, T. Tian, X.Q. Xiao, B. Zhang, Y.N. Xiao

**Affiliations:** ^1^ College of Plant Science & Technology Huazhong Agricultural University Wuhan Hubei China

**Keywords:** antagonistic activities, *Bacillus velezensis*, control efficiencies, *Fusarium oxysporum*, lotus rot

## Abstract

**Aim:**

The aim of this study was to screen potential lotus plant endophytic bacterial isolate for effective inhibition against lotus rot causing fungal pathogen *Fusarium oxysporum*.

**Methods and Results:**

In this study, endophytic bacteria were isolated from lotus tissues and tested for antagonistic activities against the pathogenic fungus *F. oxysporum*. Among the putative endophytic *Bacillus* strains identified, suspensions of the strain B‐36 showed the highest inhibition rate against *F. oxysporum* growth. Pot assays indicated that B‐36 was effective in controlling *F. oxysporum*‐inducing lotus rot. However, the control efficiency varied with the inoculation method and concentration, where injection of 800 μl B‐36 suspension per plant (2 × 10^8^ CFU per ml) into stems showed the highest control efficiencies of 77·1 and 60·0% for pre‐inoculation and post‐inoculation. In addition, the colonizing population levels (CPLs) of B‐36 on lotus also varied with the inoculation method and concentration, with the highest CPLs, that is, 3·05 and 2·83 log(CFU per gram), being observed on lotus leaves and stems respectively for stem injection of 200 μl per plant. Moreover B‐36 showed no noticeable effects on lotus seed germination rate or seedling growth. Finally, B‐36 was characterized as *Bacillus velezensis* based on its morphology, Gram‐positive characteristics, as well as its 16S rDNA and *gyrB* sequences.

**Conclusion:**

The isolate B‐36 can be applied as a biocontrol agent against *F. oxysporum*‐inducing lotus rot.

**Significance of Impact of the Study:**

The soil‐borne fungus *F. oxysporum* causes lotus rot and severe yield loss, and currently available control methods are very limited. Here we identify a new promising biocontrol agent against lotus rot caused by *F. oxysporum*.

## Introduction

Lotus (*Nelumbo nucifera*) is an aquatic plant widely cropped worldwide owing to its applications in food and drugs (Zhang *et al.* 2015; Sharma *et al.* 2017). The soil‐borne fungus *Fusarium oxysporum* is distributed worldwide with a great diversity of host‐specific strains, causing considerable damage to various crops (Gordon 2017). Lotus rot induced by *F. oxysporum* can result in the wilt of lotus plants causing significant yield loss (Rana *et al.* 2017) and postharvest damage to lotus roots (Tang *et al.* 2017).

In recent years, researchers have been paying increasing attention to fungi and bacteria, especially plant endophytes, as biocontrol agents of plant pests (Miliute *et al.* 2015; Zheng *et al.* 2016). Currently, several biological control products are available for *F. oxysporum* infecting ornamentals and crops (Lecomte *et al.* 2016; Raza *et al.* 2017), but few are available for controlling *F. oxysporum* infections in ornamentals. Additionally, a number of endophytic fungi and bacteria have been isolated from lotus plants, including the genera *Colletotrichum*, *Diaporthe*, *Rhizobium* and *Pseudomonas* (Pawlik *et al.* 2017; Chen and Kirschner, 2018). These endophytic bacteria have also been shown to possess plant growth‐promoting traits (Pawlik *et al.* 2017). For instance, *Bacillus* sp. are considered bacteria with the widest potential application in plant pest control (Wu *et al.* 2015), and some of them are endophytic bacteria with plant growth‐promoting activity (El‐Deeb *et al.* 2013; Radhakrishnan *et al.* 2017; Zhang *et al.* 2019). The production of antifungal and antibacterial compounds has been reported as the main mechanism by which *Bacillus* sp. control plant pests (Santoyo *et al.* 2012; Mora *et al.* 2015).

*Bacillus amyloliquefaciens* that has been recently classified as being *B. velezensis* is reported to have antagonistic activities against fungal and bacterial pests on plants (Ji *et al.* 2013; Dunlap *et al.* 2016; Masum *et al.* 2018; Nikolic *et al.* 2019). Its antagonistic activities were identified by genome analysis of *B. velezensis* FZB42 that was previously named as *B. amyloliquefaciens* FZB42, and four gene clusters were found to be involved in the synthesis of antibacterial compounds (Chen *et al.* 2009; Fan *et al.* 2018). However, the control efficiency of *B. velezensis* against lotus rot caused by *F. oxysporum* remains unknown.

The main objective of our work was to evaluate the potential of the endophytic *Bacillus* strain B‐36 isolated from lotus plants for the biocontrol of *F. oxysporum*‐inducing lotus rot through analysing its antagonistic activity against *F. oxysporum*‐inducing lotus rot and its colonizing ability on lotus plants.

## Materials and methods

### Isolation of lotus endophytic *Bacillus* strains

Endophytic *Bacillus* strains were isolated from healthy lotus plants in fields around Wuhan, China, according to the protocols described by Lai *et al. *(2012) with some modifications. Briefly, lotus tissues, including leaves, lotus pods, stems, rhizomes and seeds, were washed with tap water and incubated in an ultrasonic bath for 10 min, surface sterilized with 75% ethanol and 2% NaClO_3_ for 5 min each, followed by washing three times with sterilized water. The tissues were then cut into small pieces and treated with 80°C sterilized water for 10 min to enrich for *Bacillus* sp., followed by culture on nutrient agar (NA) medium at 28°C for 16 h to check surface sterilization. Finally, the surface sterilized lotus tissues were ground in 5 ml of sterilized water and sterilized quartz sand using a mortar and pestle. After incubation on NA medium at 28°C for 24 h, individual bacterial colonies were isolated and purified.

### Screening of the putative *Bacillus* strains with antifungal activities against *F. oxysporum*

The dual culture technique was applied to detect the antagonistic activities of the putative *Bacillus* strains against *F. oxysporum* that we had previously isolated from lotus with rot disease. A 3‐mm‐diameter disc from a 7‐day‐old mycelial culture of *F. oxysporum* was placed in the centre of fresh potato dextrose agar (PDA) plates (90 mm). A fresh bacterium colony was then inoculated longitudinally on the right and left sides of the fungal disc at 2 cm distance using a sterile inoculating needle. The PDA plate inoculated only with *F. oxysporum* was used as a negative control. After incubation at 25°C for 3 days, the antagonistic effect was evaluated by measuring the inhibition zones and colony diameters. The percentage of growth inhibition was calculated by the following equation: *n *= [(*A−B*)/*A*] × 100, where *A* is the colony area of uninhibited fungi and *B* is the colony area of treated fungi (Etebarian *et al.* 2005). The values were recorded as the means of four replicates, and each experiment was repeated twice.

The antifungal activity of sterilized broth filtrates from B‐36 against *F. oxysporum* was assessed as follows. Specifically, B‐36 was cultured in 4 ml nutrient broth (NB) medium at 28°C and 280 rev min^−1^ for 24 h. One millilitre of culture was transferred to 100 ml of potato dextrose broth (PDB) medium and further cultured under the same conditions for 72 h. The cell‐free supernatant was collected by centrifugation at 6000 ***g*** for 10 min, followed by filtration through 0·45 μm cellulose nitrate filters. Different broth filtrate concentrations (V_B‐36_/V_PDA_: 1, 5, 10 and 20%) were added into the PDA medium, and *F. oxysporum* was placed in the centre of fresh PDA plates as described above and cultured at 25°C. Meanwhile, sterilized water was used as a negative control. After 7 days of incubation, the colony diameters and percentage of growth inhibition of *F. oxysporum* were estimated as described above. The values were recorded as the means of three replicates, and each experiment was repeated twice.

The antifungal effect of B‐36 volatiles against *F. oxysporum* was analysed as described by Fernando *et al. *(2005) with minor modifications. Briefly, after incubation in NB medium for 24 h, 200 μl of B‐36 suspension was plated on one half of a divided plate containing PDA medium, and *F. oxysporum* was inoculated on the other half, followed by resealing the plates and culturing at 25°C. Meanwhile, plates without B‐36 volatiles were used as a negative control. After 7 days of incubation, the growth inhibition percentage of *F. oxysporum* was estimated as described above, and its mycelial morphology was observed through an optical microscope. There were three replicates for each treatment, and each experiment was repeated twice.

### Assessment of biocontrol activity of B‐36 against lotus rot caused by *F. oxysporum*

Similar‐sized healthy rhizomes of lotus (*N. nucifera*) cultivar Taikong 6 were transferred into plastic pots (30 × 15 cm, diameter × height) filled with sterilized soil (one rhizome per pot). The lotus plants were cultured at approximately 25°C in a greenhouse and plants showing the appearance of the first leaf were used in the following experiments. Briefly, B‐36 and the pathogen *F. oxysporum* were cultured on PDB and PDA medium respectively. B‐36 cultures and *F. oxysporum* spores were collected by centrifugation at 8000 ***g*** for 5 min, followed by three washes with sterilized water. Finally, 2 × 10^8^ CFU per ml B‐36 and 1 × 10^4^ spores per ml *F. oxysporum* spore suspension were prepared as described by Lin *et al. *(2018).

To analyse control efficiency with different inoculation methods and concentrations, B‐36 was inoculated on lotus plants by foliar spraying or injecting 200, 400 and 800 μl B‐36 suspension per plant into stems. *F. oxysporum* was inoculated by injecting 100 μl spore suspension per plant into stems. B‐36 control efficiencies on *F. oxysporum*‐inducing lotus rot were analysed in terms of pre‐ and post‐inoculation with different inoculation methods and concentrations. In this study, B‐36 pre‐inoculation refers to the inoculation of B‐36 on lotus plants 15 days before the inoculation of *F. oxysporum*, whereas B‐36 post‐inoculation refers to the inoculation of B‐36 7 days post‐inoculation (dpi) of *F. oxysporum* on lotus plants. Corresponding positive and negative controls were set up by spraying the fungicide carbendazim on the leaves and by injecting 800 μl of sterilized water into stems per plant respectively. There were six replicates and two biological replicates for each treatment.

The disease indices of each plant were measured at 30, 60 and 90 dpi of the second microbe on lotus plants. Disease severity was classified as 0, 1, 3, 5, 7 and 9 depending on the diseased leaf area (0, <10, 10–25, 25–50, 50–75 and >75% of the total respectively). The disease index (DI) and control effects (Ce) for the pathogen were calculated by the following equation:

$$\text{DI}\;=\;[\sum \left(\text{scale}\;\times \;\text{number}\;\text{of}\;\text{plants}\;\text{infected}\right)/\left(\text{highest}\;\text{scale}\;\times \;\text{total}\;\text{number}\;\text{of}\;\text{plants}\right)]\;\;\times \;100$$

$$\text{Ce}\;\left(\%\right)\;=\;\left[\left({\text{DI}}_{\text{Control}}-{\text{DI}}_{\text{Test}}\right)/{\text{DI}}_{\text{Control}}\right]\;\times \;100.$$

### Analysis of endophytic colonization of B‐36 in lotus

B‐36 was acclimated to the different concentrations of the antibiotic rifampicin (Sigma, R3501, St. Louis, MO) by continuous culture at 28°C for 24 h on NA medium, that is, 1, 5, 25, 50, 100, 200 and 400 μg ml^−1^ rifampicin. The acclimated strain B‐36^R^ that could grow on NA medium containing 400 μg ml^−1^ rifampicin was collected for further culture on PDB medium containing 400 μg ml^−1^ rifampicin at 28°C and 160 rev min^−1^ for 16 h. Finally, a 2 × 10^8^ CFU per ml B‐36^R^ suspension was obtained as described above.

To test B‐36^R^ colonization efficiencies on lotus tissues, lotus plants were inoculated with B‐36^R^ suspension by three different methods: (i) soaking seeds for 10 min, (ii) foliar spraying of B‐36^R^ suspension on lotus plants with one leaf, and (iii) injecting 100 and 200 μl B‐36^R^ suspension per plant into stems of the lotus plants with one leaf. For each experiment, sterilized water was used as a negative control. After co‐cultivation at 25°C in a greenhouse for different periods of time (1, 7, 15, 30 and 45 days), the colonization efficiencies of B‐36^R^ on lotus leaves and stems were analyzed for each time point. B‐36^R^ colonies in inoculated lotus leaves and stems were isolated as follows. Briefly, approximately 3 g of fresh leaves and stems were weighed and ground separately in 5 ml sterilized water. Thereafter, 100 μl of each tissue suspension was diluted, plated on NA medium containing 400 μl ml^−1^ rifampicin, and cultured at 28°C for 24 h. Finally, the number of B‐36^R^ colonies on each plate was counted, and B‐36^R^ population densities were evaluated in lotus stems and leaves. There were three technical replicates and two biological replicates for each treatment.

### Evaluation of B‐36 on lotus seed germination and growth

Similar‐sized healthy lotus seeds were washed with tap water, followed by two washes with sterilized water and soaking the seeds in B‐36 suspension (2 × 10^8^ CFU per ml) for 1 h, using seeds soaked in sterilized water as a negative control. All the seeds were sown in sterilized sandy soils and cultured at 25°C in a 16 h light/8 h dark cycle. After 15 days of co‐cultivation, the seed germination rate, average height and above‐ground biomass were evaluated. There were seven individual plants and two biological replicates for each test group.

### Identification of B‐36

Gram staining of B‐36 was performed according to the method described by Preston and Morrell (1962). Briefly, 16S rDNA and *gyrB* were amplified using specific primer pairs, 5′‐AGAGTTTGATCCTGGCTCAG‐3′ and 5′‐ACGGCTACCTTGTTACGACT‐3′, and 5′‐GAAGTCATCATGACCGTTCTGCAYGCNGGNGGNAARTTYGA‐3′ and 5′‐AGCAGGGTACGGATGTGCGAGCCRTCNACRTCNGCRTCNGTCAT‐3′ respectively (Weisburg *et al.* 1991; Peng 2013). PCR assays were performed in a reaction mixture containing 1 μl B‐36 suspension, 10 mmol l^−1^ Tris‐HCl, 50 mmol l^−1^ KCl, 100 μmol l^−1^ of each dNTP, 1 μmol l^−1^ of each primer, 2·5 mmol l^−1^ MgCl_2_ and 2·5 U of *Taq* DNA polymerase. For 16S rDNA, thermal cycler conditions consisted of a denaturation step (5 min at 94°C), followed by 30 cycles of denaturation at 94°C for 30 s, annealing at 56°C for 30 s and extension at 72°C for 1 min. For *gyrB*, thermal cycler conditions consisted of a denaturation step (5 min at 94°C), followed by 30 cycles of denaturation at 94°C for 40 s, annealing at 60°C for 40 s and extension at 72°C for 2 min.

The sequences of closely related *Bacillus* strains were obtained from GenBank (https://www.ncbi.nlm.nih.gov/; for *gyrB*) and RDP (https://rdp.cme.msu.edu/; for 16S rDNA) databases.

### Statistical analysis

All data were expressed as mean ± SE. The experimental data were analysed using standard analysis of variance (anova) followed by Duncan’s multi‐range test. Values with *P* < 0·05 were considered statistically significant. anova was conducted using spss software, ver. 30.

## Results

### Endophytic bacterial strain B‐36 isolated from lotus showed high antagonistic activity against *F. oxysporum*

**Figure 1:**
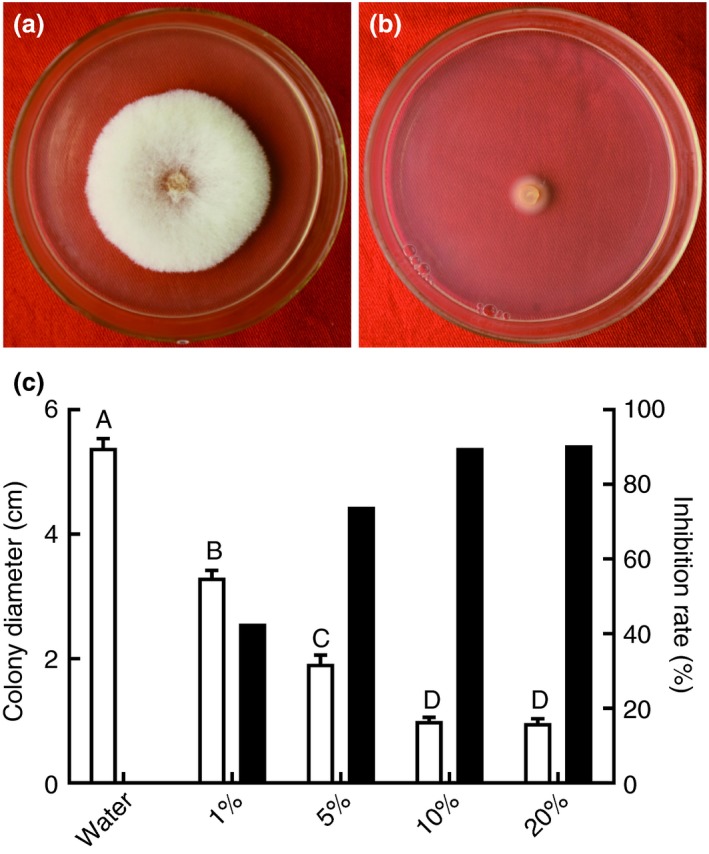
Antagonistic activities of B‐36 broth filtrates against *Fusarium oxysporum* at different concentrations. Sterilized water was used as a negative control (a), and *F. oxysporum* was cultured on PDA with various concentrations of B‐36 broth filtrate (b) at 25°C for 7 days. The colony diameters and inhibition rates of *F. oxysporum* were recorded (c) (□ colony diameter; ■ inhibition rate). Values are means ± SE, *n* = 3. Different letters above the bars denote significant differences at *P* < 0·01 according to Duncan’s multi‐range test. [Colour figure can be viewed at https://www.wileyonlinelibrary.com]

Of all the endophytic bacterial strains isolated from lotus, 10 strains exhibited antagonistic activities against *F. oxysporum* (Table 1). Among these 10 strains, strain B‐36 showed the highest antifungal activity against *F. oxysporum*, with a mycelial growth inhibition rate of 74·1% (Table 1). In addition, B‐36 broth filtrates displayed a strong antifungal activity against *F. oxysporum* in a dose‐dependent manner, with mycelial growth inhibition rates of 42·7 and 90·5% for 1 and 20% B‐36 broth filtrates respectively (Fig. 1). However, for B‐36 volatiles, no significant antifungal activities against *F. oxysporum* were observed (data not shown).

**Table 1:** Antagonistic activities of different bacterial strains against *Fusarium oxysporum*

Strains	Average colony diameter (mm)*	Inhibition rate (%)
Control†	54·0^a^	–
B‐36	14·0^b^	74·1
B‐22	15·0^bc^	72·2
B‐5	15·9^cd^	70·4
B‐2	17·0^de^	68·5
B‐30	17·5^de^	67·6
B‐34	18·0^ef^	66·7
B‐19	18·5^efg^	65·7
B‐23	19·5^fg^	63·9
B‐10	20·0^g^	63·0
B‐35	20·1^g^	62·9

* Different letters indicate a significant difference at *P* < 0·05 according to Duncan’s multi‐range test.

† *F. oxysporum* cultured on PDA plates were used as the control.

### B‐36 has high biocontrol efficiency against lotus rot caused by *F. oxysporum*

**Figure 2:**
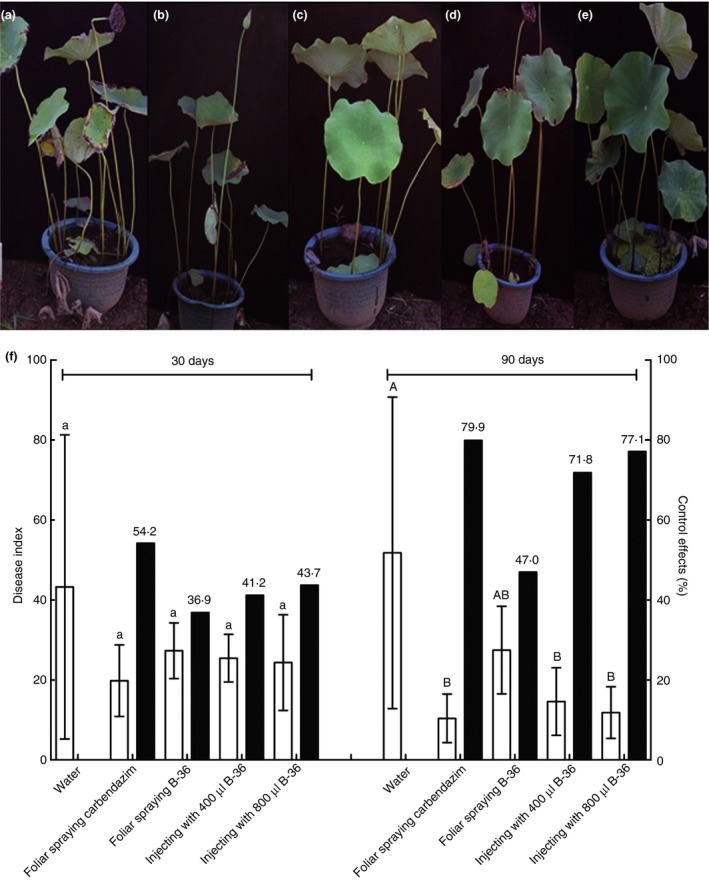
Control efficiencies of B‐36 pre‐inoculation on lotus rot caused by *Fusarium oxysporum*. Negative and positive controls were set up by injecting sterilized water into lotus stems (a) and spraying carbendazim on lotus leaves (b) respectively. B‐36 was pre‐inoculated on lotus plants as follows: foliar spraying of B‐36 suspension (c) and injecting 400 (d) and 800 μl B‐36 suspension (e) per plant into stems. At 15 dpi, the pathogen *F. oxysporum* was inoculated on all the lotus plants tested. Growth status and disease index of lotus plants with different treatments were investigated at 30 and 90 dpi of *F. oxysporum* (f) (□ disease index; ■ control effects). Values represent means ± SE, *n* = 6. Different letters above the bars denote a significant difference at *P* < 0·05 and *P* < 0·01 according to Duncan’s multi‐range test. [Colour figure can be viewed at https://www.wileyonlinelibrary.com]

**Figure 3:**
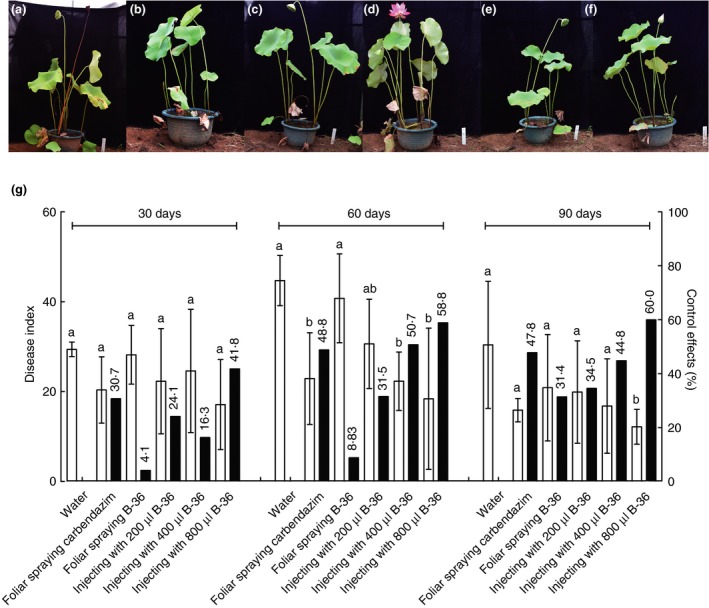
Control efficiencies of B‐36 post‐inoculation on lotus rot caused by *Fusarium oxysporum*. The pathogen *F. oxysporum* was pre‐inoculated on all the lotus plants tested. Negative and positive controls were set up by injecting sterilized water into lotus stems (a) and spraying carbendazim on lotus leaves (b), respectively. At 7 dpi, B‐36 was inoculated on lotus plants as follows: foliar spraying of B‐36 suspension (c) and injecting 200 (d), 400 (e), and 800 μl B‐36 suspension (f) per plant into stems. Growth status and disease index of lotus plants with different treatments were investigated at 30, 60, and 90 dpi of B‐36 (g) (□ disease index; ■ control effects). Values are means ± SE, *n* = 6. Different letters above the bars denote a significant difference at *P* < 0·05 according to Duncan’s multi‐range test. [Colour figure can be viewed at https://www.wileyonlinelibrary.com]

Pot experiments were designed to evaluate the biocontrol efficiency of strain B‐36 against lotus rot caused by *F. oxysporum*. At 90 dpi, pre‐inoculation B‐36 biocontrol efficiencies against lotus rot were 71·8 and 77·1% after injecting 400 and 800 μl B‐36 per plant into stems respectively compared to no significant biocontrol effect by foliar spraying of B‐36 (Fig. 2). In addition, significant post‐inoculation biocontrol efficiencies of B‐36 against lotus rot were also recorded for stem injection of 400 and 800 μl per plant at 60 dpi and 800 μl per plant at 90 dpi, that is, 50·7, 58·8 and 60·0% respectively (Fig. 3). No apparent biocontrol against lotus rot was observed for any inoculation method at 30 dpi for B‐36 pre‐inoculation and post‐inoculation (Figs 2 and 3).

### B‐36 can successfully colonize lotus

**Figure 4:**
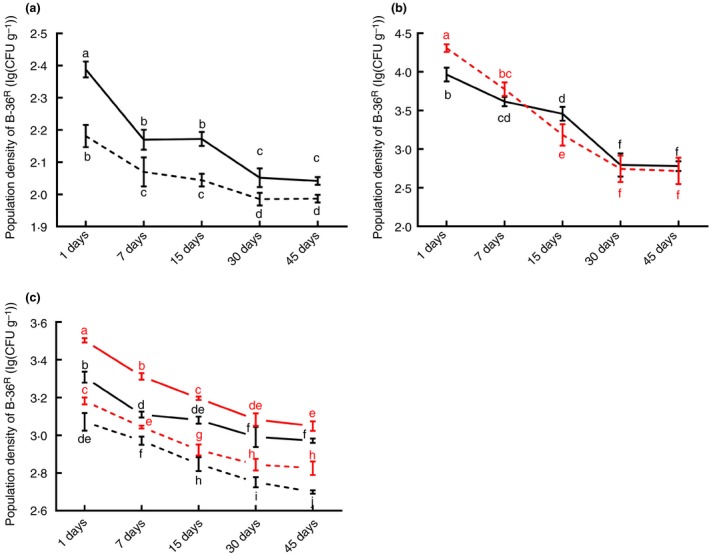
Population dynamics of B‐36^R^ in lotus tissues after different inoculation methods. Using the acclimated rifampicin‐resistant strain B‐36^R^ as the target bacterium, colonizing population levels of B‐36^R^ in both lotus stems (black) and leaves (red) were detected. B‐36^R^ was inoculated on lotus plants as follows: soaking seeds in B‐36^R^ suspension (a: 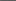 Stems; 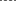 Leaves), foliar spraying of B‐36^R^ suspension (b: 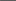 Stems; 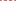 Leaves), and injecting 100 (solid) and 200 μl (dashed) B‐36^R^ suspension per plant into stems (c: 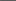 100 μl: Stems; 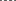 100 μl: Leaves; 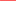 200 μl: Stems; 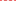 200 μl: Leaves). Sterilized water was used as a negative control. Values represent means ± SE, *n* = 3. Different letters above the bars denote a significant difference at *P* < 0·05 according to Duncan’s multi‐range test. [Colour figure can be viewed at https://www.wileyonlinelibrary.com]

To evaluate B‐36 colonization in lotus, the rifampicin‐resistant strain B‐36^R^ was developed. Results indicated that B‐36^R^ could grow well in NA medium containing 400 μg ml^−1^ rifampicin, which showed that B‐36^R^ obtained high resistance to rifampicin (Fig. 4a). Therefore, the strain B‐36^R^ was used to study colonization dynamics in lotus plants. Results showed that B‐36^R^ could successfully colonize lotus leaves and stems via three different inoculation methods, that is, seed soaking, foliar spraying and stem injection (Fig. 4b–d). For each inoculation method, B‐36^R^ CPL was significantly decreased at 30 dpi; however, it was higher in stems than in leaves except for foliar spraying (Fig. 4b–d). In addition, among three inoculation methods, the highest B‐36^R^ CPL was observed in stems and leaves at approximately 3·05 and 2·83 log(CFU per gram) respectively at 30 dpi of treatment with 200 μl suspension per plant. In contrast, the lowest CPLs in the stems and leaves were around 2·04 and 1·99 log(CFU per gram) after soaking seeds with B‐36^R^ (Fig. 4b–d). No B‐36^R^ colonies were isolated in any of the negative controls.

### B‐36 showed no apparent effects on lotus seed germination and seedling growth

Compared to the control seeds, soaking seeds with B‐36 suspension showed no significant change (*P *> 0·05) in seed germination rate (approximately 86·4%) (Fig. S1). It also had no apparent effects on the height and above‐ground biomass of 15‐day‐old seedlings, which were approximately 18·61 cm and 4·73 g respectively (Fig. S1).

### B‐36 was characterized as *Bacillus velezensis*

The colony of strain B‐36 on NA medium was opaque, white and dry (Fig. S2a). In addition, B‐36 was characterized as a Gram‐positive bacterium by Gram staining with the typical characteristics of the genus *Bacillus* (Fig. S2b). This was further confirmed by 16S rDNA (GenBank accession no. MK182731) and gyrB‐encoding gene (GenBank accession no. MK185102) sequence analyses, which were 99·9 and 99·4% identical respectively to the known sequences of *B. amyloliquefaciens* (AB244285 in RDP database and JN412504 in GenBank database) (data not shown). Based on these data, B‐36 belongs to *B. amyloliquefaciens* that has recently been classified as *B. velezensis*.

## Discussion

*Fusarium oxysporum* is a soil‐borne pathogen that causes severe damage in a number of crops (Dean *et al.* 2012). Recent data indicate an expanded host range of *F. oxysporum* (Matic *et al.* 2018), implying the urgent need of effective management strategies. Several biological agents against plant wilt diseases caused by *F. oxysporum* have been identified, including plant endophytic fungi and bacteria (Lecomte *et al.* 2016; Raza *et al.* 2017; Hajji‐Hedfi *et al.* 2018).

Among bacteria, *Bacillus* sp. are believed to have the highest potential for pest biocontrol (Shafi *et al.* 2017). As a *Bacillus* sp. strain, *B. velezensis* has been reported to be effective in controlling *F. oxysporum‐*caused wilt disease in tomato (Elanchezhiyan *et al.* 2018), indicating its potential as a pest biocontrol agent on other crops. In this study, an endophytic bacterium, *B. velezensis* strain B‐36, was isolated from lotus plants, which showed high antifungal activity against *F. oxysporum*. B‐36 appears to inhibit *F. oxysporum* growth mainly through the production of antifungal agents, as its broth filtrates showed relatively high antagonistic activity against *F. oxysporum*. Previous studies have also identified antifungal compounds synthesized by *B. velezensis*, such as peptides and lipopeptides (Romano *et al.* 2011; Alvarez *et al.* 2012; Kim *et al.* 2015; Luna‐Bulbarela *et al.* 2018).

Pot assays showed that B‐36 had significant control efficiency against lotus rot caused by *F. oxysporum*. The control efficiency was closely related to B‐36 concentration and inoculation method. Injecting over 400 μl B‐36 per plant into the stems could result in a significant control efficiency against lotus rot, probably owing to a higher B‐36 CPL in lotus plants. In contrast, a lower B‐36 CPL from foliar spraying resulted in no obvious control efficiency against lotus rot. Moreover B‐36 controls lotus rot in a dose‐dependent manner, implying that its significant control efficiency against lotus rot could be mainly achieved by antifungal compounds rather than by priming lotus defenses, despite several reports on the induction of the plant defense response by *B. velezensis* (Yamamoto *et al.* 2015a; Yamamoto *et al.* 2015b). Meanwhile, the detection of remarkable B‐36 control efficiencies against lotus rot at later stages post‐B‐36 inoculation rather than at the early stage might be caused by persistent inhibition of *F. oxysporum* growth by B‐36, as well as higher disease tolerance of lotus plants at late developmental stages. It is worth noting that injecting 800 μl B‐36 per plant into stems showed significantly higher control efficiency against lotus rot than the fungicide carbendazim, indicating the promising potential of B‐36 as a biological agent against *F. oxysporum* on lotus.

In conclusion, *B. velezensis* strain B‐36 could serve as a potential biocontrol agent against *F. oxysporum* in lotus plants due to its significant biocontrol properties and colonizing ability on lotus plants, as well as the lack of apparent negative effects on lotus seed germination and seedling growth.

## Conflict of Interest

None.

## Supporting information


**Figure S1**. Effects of B‐36 on lotus seed germination and seedling growth.

**Figure S2**. Gram staining and microscopic examination of B‐36.
