# Supplementary figures and images for: Endophytic Bacillus velezensis strain B‐36 is a potential biocontrol agent against lotus rot caused by Fusarium oxysporum
Source: J Appl Microbiol. 2019 Dec 19;128(4):1153–62. doi: 10.1111/jam.14542 (PMC7079251; doi:10.1111/jam.14542)

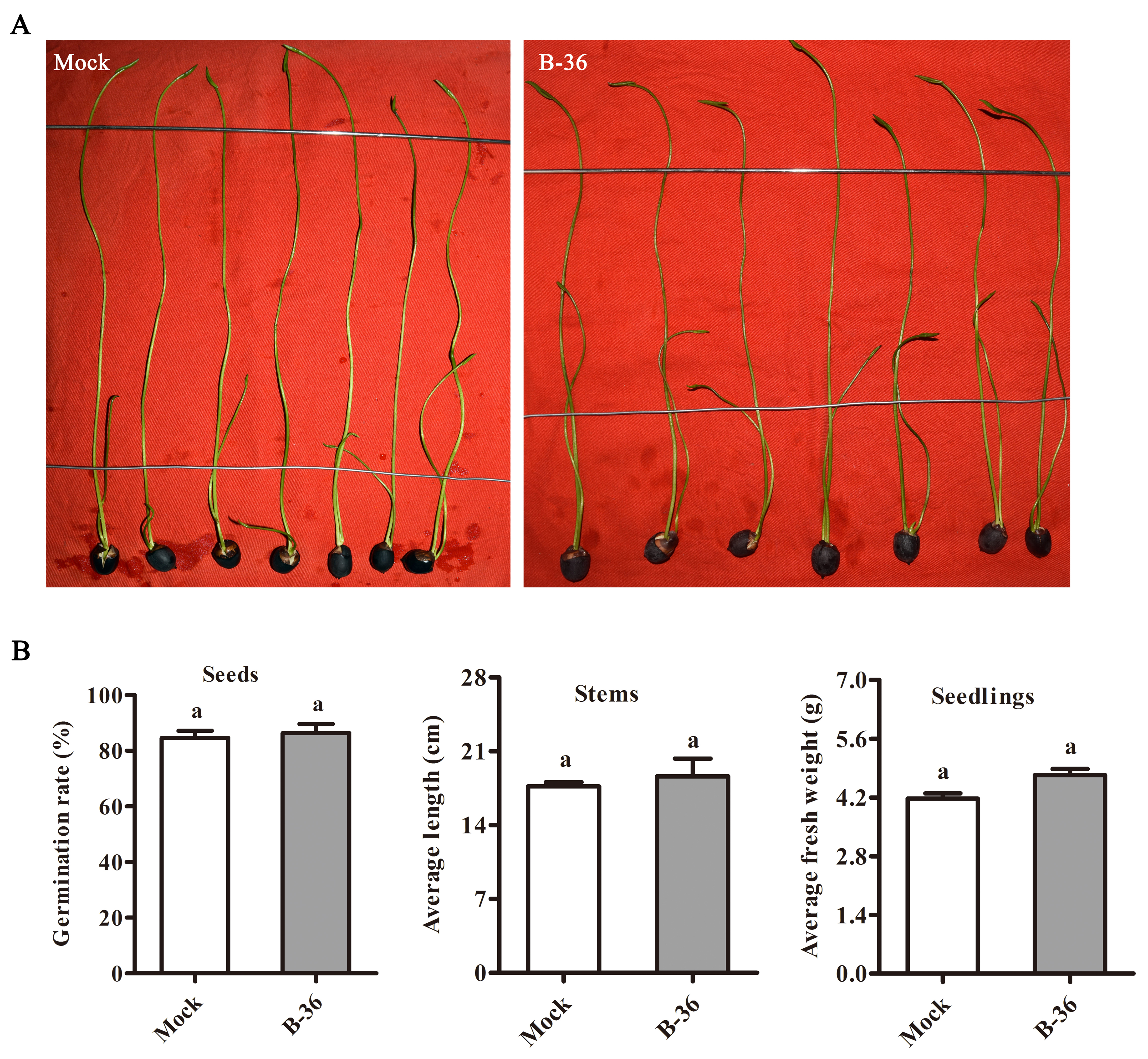

Supplement: Supplementary file 1 — Figure S1. Effects of B‐36 on lotus seed germination and seedling growth. [file JAM-128-1153-s001.jpg]

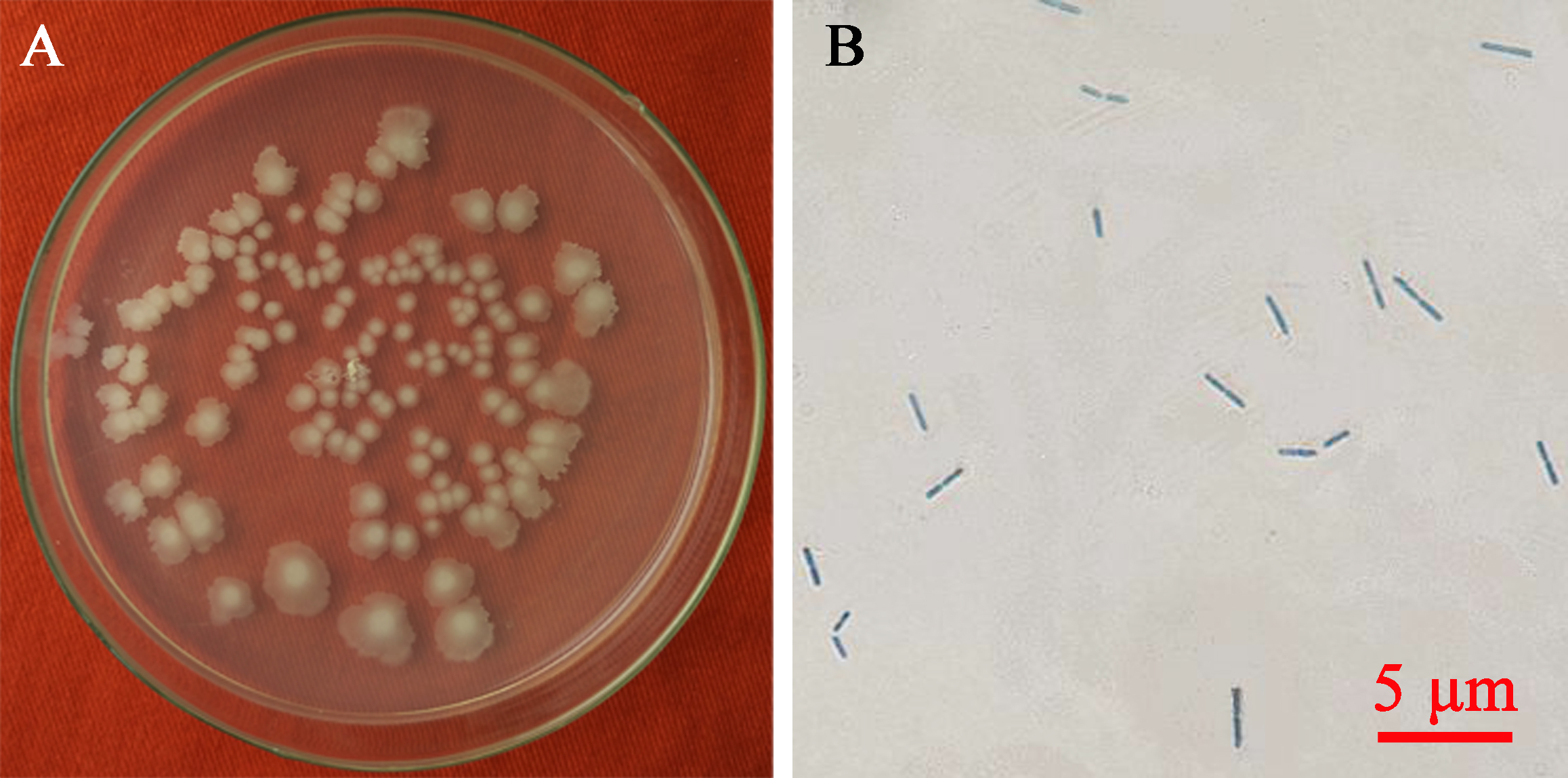

Supplement: Supplementary file 2 — Figure S2. Gram staining and microscopic examination of B‐36. [file JAM-128-1153-s002.jpg]
